# Insights and Trends in Open Note Access: Retrospective Observational Study

**DOI:** 10.2196/55982

**Published:** 2024-12-17

**Authors:** Randeep Singh Badwal, Paul Cavo, Mandip Panesar

**Affiliations:** 1 Department of Biomedical Informatics University at Buffalo Buffalo, NY United States; 2 Erie County Medical Center Buffalo, NY United States

**Keywords:** open note trends, open notes, open note access, open note use, open note sex, open note specialty, clinical note views, patient portal, patients, trends, hospitals, engagement, retrospective observational study, outpatient, assessment, older patients, adults, pandemic, COVID-19

## Abstract

**Background:**

As of 2021, at least 4 out of every 5 hospitals offered patients access to clinical notes via a web-based patient portal, a number that is expected to grow because of the 21st Century Cures Act. There is limited data on how open note use may have evolved over time or which types of clinical interactions were viewed most in the outpatient setting.

**Objective:**

This study aims to analyze trends in outpatient open note access over time; characterize usage in terms of age, sex, and clinical interaction type; and assess the method of access to help uncover areas of improvement in patient engagement and identify further areas of research.

**Methods:**

A retrospective observational study was conducted at Erie County Medical Center from November 1, 2021, to December 31, 2022, to coincide with the time that open notes went live. Outpatient note access and account logs were downloaded from the portal and combined into a single dataset consisting of 18,384 note accesses by 4615 users, with column headings of the patient index, sex, age, note title that was accessed, clinical interaction type, time stamp of note creation, time stamp of access, and method of access (web vs mobile). A separate table was created with sex data for all 35,273 portal accounts. Microsoft Excel and Microsoft Power Query were used to combine and analyze the data.

**Results:**

During the study period, 4615 portal users viewed 12,150 documents for a total of 18,384 times, averaging 2.6 notes per patient viewed 4 times. Only 13.1% (4615/35,273) of all portal inpatient and outpatient registrants viewed their outpatient notes. There was a female predominance in those who viewed notes (2926/4615, 63.4%; *P*<.001), while 56.8% (20,047/35,273) of all portal registrants were female. Users in their 30s and 50s accessed more notes than other age groups. The ratio of mobile-to-web access of notes tended to decrease as a function of increasing age, which was not observed in those aged ≥90 years. Notes regarding COVID-19 assessments were the most accessed among all clinical interactions (4725/12,150, 38.9%). Overall, the number of users accessing notes reached a maximum of 1968 before declining to 1027 by the end of the study period.

**Conclusions:**

Open note access was largely dominated by COVID-19 assessments, and the number of users viewing their notes has declined over time as the pandemic subsided. Furthermore, female patients and those aged in their 30s as well as 50s viewed more notes than other groups. Finally, the percentage of notes viewed via a mobile device tended to decrease as a function of increasing age, showing that web-based access of open notes is an important modality for older patients.

## Introduction

Medical notes are comprehensive narratives generated by health care providers during encounters with patients. They form an important backbone of the medical record since they summarize the problem, diagnosis, and treatment plan for various ailments that a patient may experience throughout their lifetime. As of 2021, a total of 78% of office-based physicians and 96% of nonfederal acute care hospitals have implemented a certified electronic health record (EHR) into practice [1], which affords the ability to document these notes digitally. Although previously it was at the discretion of health care institutions to share medical notes with patients, the 21st Century Cures Act’s Final Rule [2] implemented in April 2021 mandates that all electronic health information, including notes, be made accessible to patients. This concept, referred to as “open notes” [3], is thought to foster greater transparency between a patient and their health care team, and has been shown to make patients feel more in control of their care [4] and increase medication adherence [5].

As of 2021, more than 4 out of 5 hospitals offered patients access to clinical notes via a web-based patient portal [6], a number that is expected to rise because of legislation. However, despite its widespread availability, literature published specifically on open note access remains relatively scarce. Sangal et al [7] showed that patients who visit the emergency department were less likely to read notes if they were under the age of 18 years; male; Black non-Hispanic, Hispanic, or Latino; admitted; Spanish speaking; or on public insurance. Blok et al [8] showed that patients who viewed their outpatient notes were younger; were mostly White; were mostly female; had better income status; had slightly higher rates of primary care visits; and were higher users of secure messaging than nonusers. However, how open note access may have evolved over time or which type of clinical interactions were accessed the most are worthwhile questions to explore.

For this study, we decided to focus on the outpatient rather than the inpatient setting due to differences in motivation for accessing notes in each setting. For example, there is a greater emphasis on self-care in the outpatient setting, which may motivate patients to obtain a deeper understanding of their treatment plan via a medical note, as opposed to the hospital where health care interactions are continuous. Due to the potential for such motivations to influence the number of patient interactions with the portal, we concentrated on outpatient notes rather than looking at all notes as a conglomerate. In particular, we sought to study trends in access over time; characterize access in terms of age, sex, and type of clinical interaction; and assess the method of access to help uncover areas of improvement in patient engagement as well as identify further areas of research.

## Methods

### Setting

The study was conducted at Erie County Medical Center, a level 1 regional trauma and teaching center with 573 inpatient beds and 24 practice locations in Buffalo, New York. There were approximately 154,501 visits in 2023. At registration, all patients are given the option of signing up for the Follow My Health Patient Portal, which provides the ability to request appointments and medication refills; message a provider; record health data such as blood pressure; view medical history and results; and, as of November 2021, view open notes.

### Study Design

A retrospective observational study was performed using patient portal audit data. The study period was 14 months (from November 1, 2021, to December 31, 2022).

The data were retrieved from Follow My Health, a system that administers the portal to patients who have their data stored within the EHR. On the administrative side, audit logs in .CSV format could be downloaded and thereby analyzed to inform trends about portal use. We were particularly interested in the “Health Record Viewed” audit file, which records the access of clinical documents by patient username, and the “Connected Patients” file, which lists the demographic information for each username in the portal. The portal contained a total of 35,273 accounts as of January 12, 2023.

Since we were particularly interested in the outpatient setting, the “Health Record Viewed” file was filtered by outpatient documents at the time of download, and a list of unique note titles was curated. Some titles included in this initial dataset did not correspond to notes authored by health care professionals, so we compared a list of note titles present prior to November 2021 to those that appeared after open notes went live. This led to a final list of titles that was verified with the IT team to consist only of outpatient clinical notes. The process allowed us to identify 18,385 outpatient notes that were accessed by patients and authored by health care workers, which included physicians, nurses, advanced practice providers, dieticians, and social workers. The note titles were subsequently grouped and labeled with a clinical interaction type. For example, titles such as “Internal Medicine Follow Up” and “Patient Portal Family Med Note” were grouped under “FM, IM, or primary care.” Notable absences were psychiatry notes that were documented in the inpatient EHR, as well as obstetrics and gynecology, which operate as an external entity to the institution.

During the process of labeling, we compared time stamps of document creation and discovered that a particular note may be uploaded as multiple distinct notes in the patient portal. For example, there were 1643 infectious disease (ID) notes that had identical creation time stamps with COVID-19 notes, and a sample of these notes showed that they were identical. The ID notes were relabeled as COVID-19. In addition, there were instances of duplicate notes with similar titles and other notes forming a subsection of larger notes. Although not exhaustive, 2 examples of these included “patient portal COVID progress note” and “COVID progress note,” or “annual wellness visit” and “patient portal health maintenance note.” There were 179 pairs, 2 triplets, and 1 quadruplet sets of notes that were accessed by users (1829 in total including the COVID-19 or ID notes mentioned earlier). Since a patient may access 1 version of a note while overlooking another, or they may engage with different versions on distinct dates, we chose to retain duplicate notes to capture the complete range of patient interactions with the portal.

Ultimately, the data were merged within Microsoft Power Query to obtain a single dataset for analysis, consisting of username, patient first and last names, sex, date of birth, note title that was accessed, type of clinical interaction, time stamp of note creation, time stamp of access, and method of access (web vs mobile). Demographic information of each username within the portal was carefully reviewed against the EHR to fill in missing information, uncover multiple usernames for a single patient who may have registered more than once, or identify multiple usernames for patients who had name changes but were indeed a single entity. This was done to obtain an accurate account of unique users who accessed their notes. Only 1 patient who accessed a single note (bariatrics) was deleted from the dataset because they could not be uniquely identified from the EHR, resulting in 18,384 total entries. Date of birth values were converted into age in years and all patient identifiers were removed to obtain a final dataset. A separate, single-column table was derived from the “Connected Patients” file that only listed the sex of each patient connected to the portal. Pivot tables and charts within Microsoft Excel were used to analyze the data. A chi-square test was used to compare the proportion of female patients versus male patients accessing their notes.

### Ethical Considerations

The study was approved by the institutional review board (IRB) at the University at Buffalo (IRB ID STUDY00006926) and the Research Department at Erie County Medical Center. As part of the approval, the IRB granted a full waiver of individual authorization to use protected health information for research purposes in accordance with the Health Insurance Portability and Accountability Act (HIPAA). All data were deidentified, securely stored, analyzed, and managed in compliance with regulations. There was no compensation.

## Results

**Figure 1:**
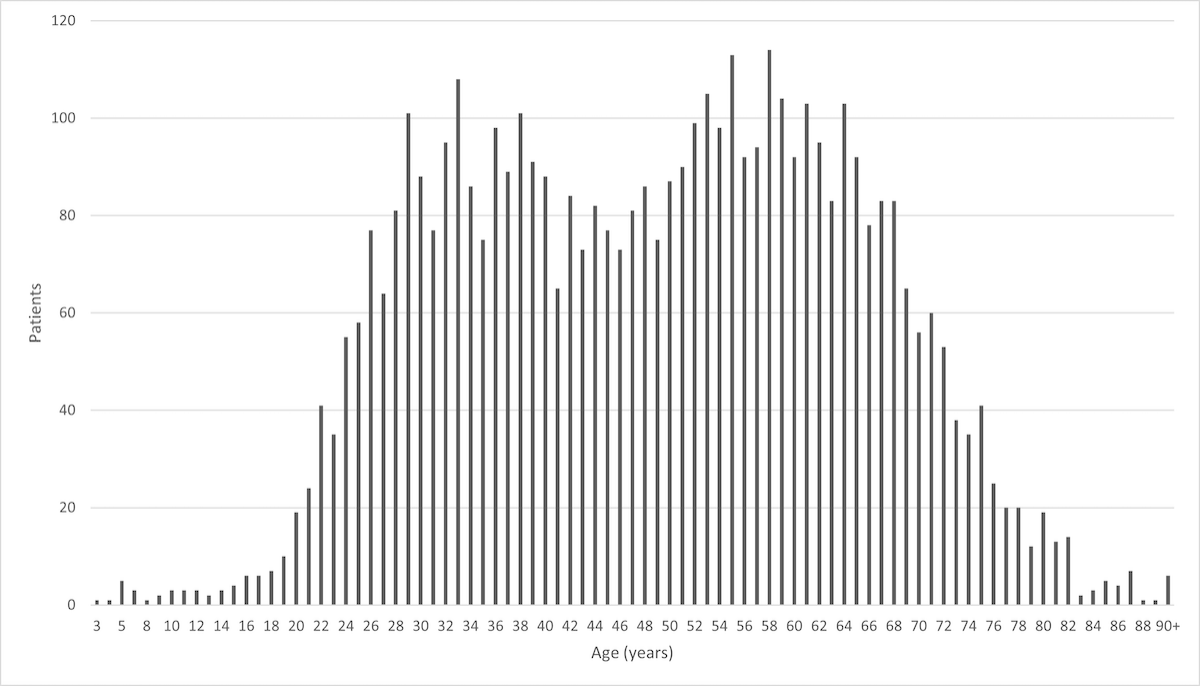
Age distribution of open note users. A bimodal peak is observed in the age range of 30s and 50s.

**Figure 2:**
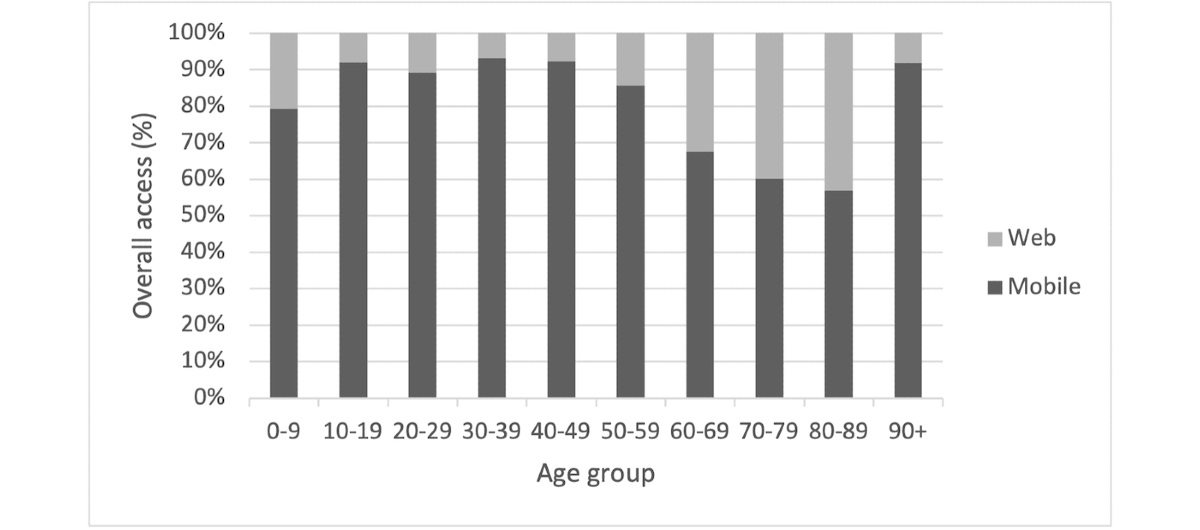
Ratio of mobile-to-web access per age group. A gradual decline in mobile use relative to web use is seen with progressing age, although this trend did not extend to those aged ≥90 years.

From November 1, 2021, to December 31, 2022, a total of 13.1% (4615/35,273) of all portal registrants viewed outpatient notes, with the denominator consisting of both inpatient and outpatient registrants. The total number of notes accessed was 18,384, out of which 12,150 were unique. This equates to each user reading 2.6 unique notes a total of 4 times. More female patients viewed notes than male patients (2926/4615, 63.4%; *P*<.001). Of all portal accounts, 56.8% (20,047/35,273) were female. In terms of age, more people in their 30s and 50s viewed notes compared to other groups (Figure 1). Approximately 82.7% (15,196/18,384) of all notes were accessed via a mobile device, a trend that tended to decrease with progressing age but was not observed in those aged 90 years and older (Figure 2).The distribution of open note accesses by clinical interaction type is shown in Table 1. The “COVID-19” category had the most instances of notes being accessed. This was followed by “family medicine, internal medicine, or primary care” with the remainder being clinical interactions from other specialties. There were 565 documents that were uncategorized since multiple specialties shared a single note title in these circumstances.

**Figure 3:**
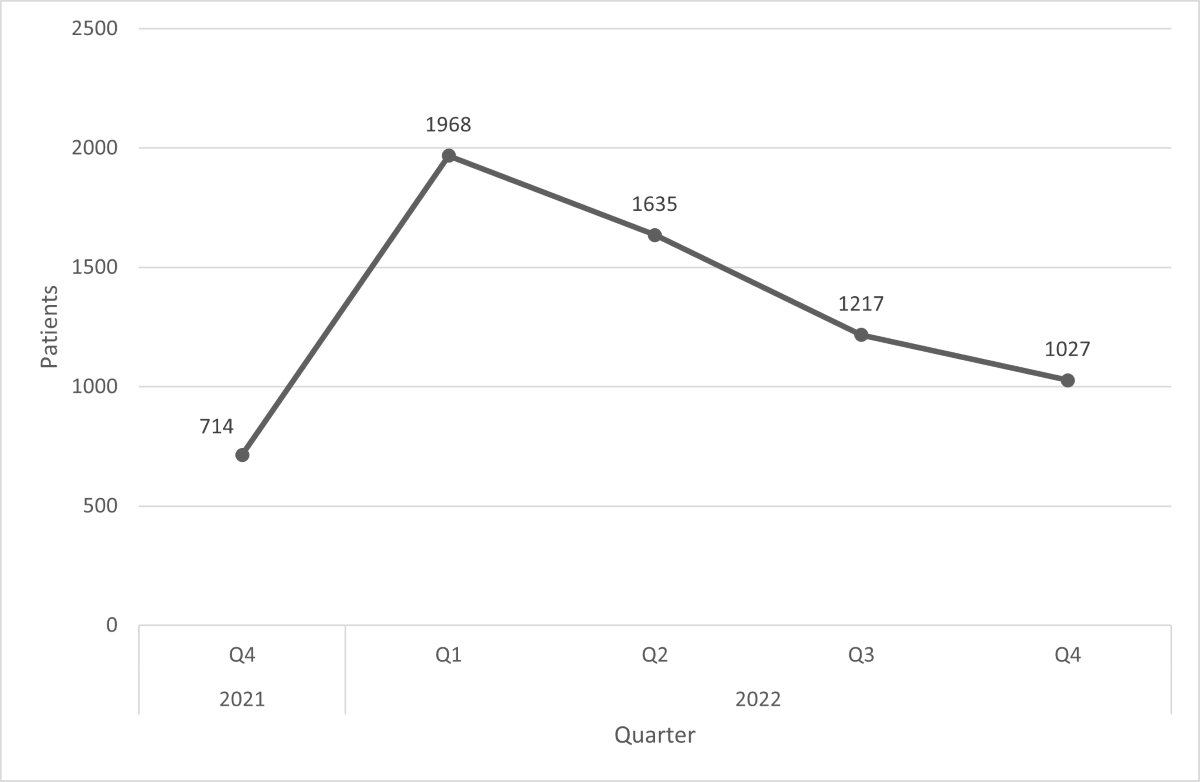
Number of patients viewing open notes each quarter (COVID-19 notes included). Q: quarter.

**Figure 4:**
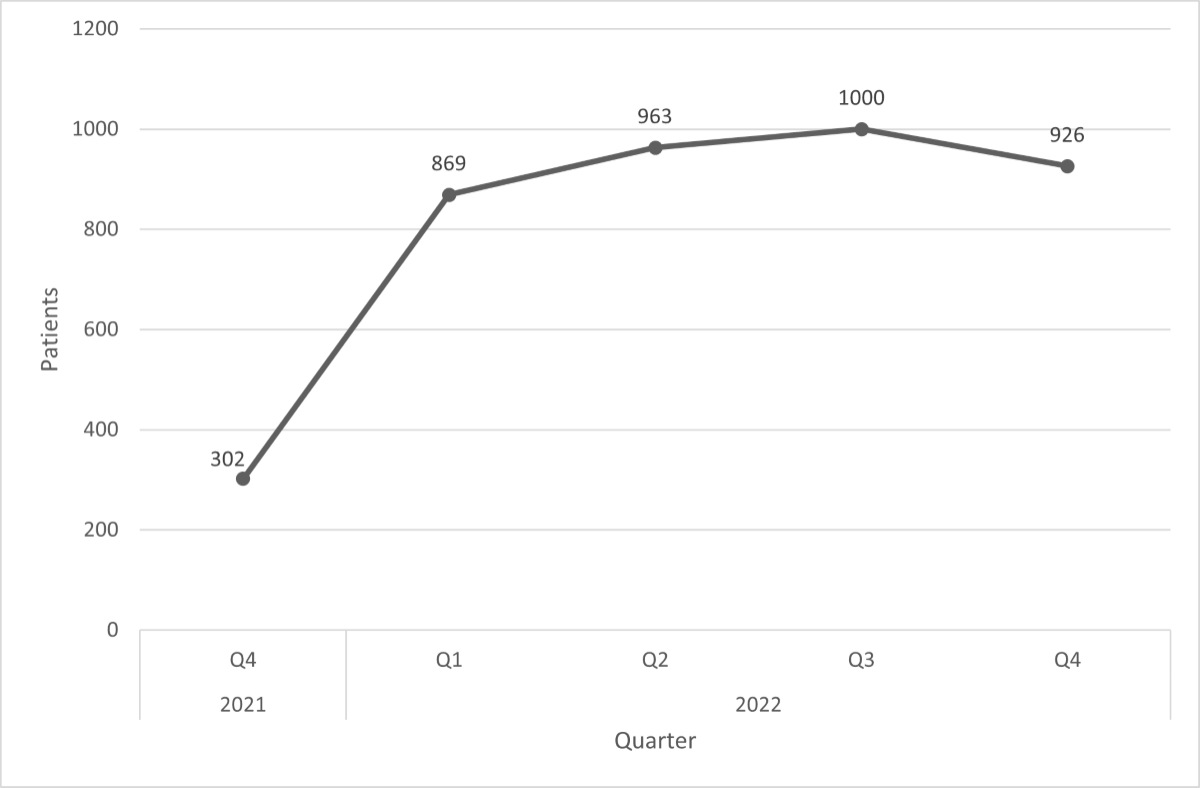
Number of patients viewing open notes each quarter (COVID-19 notes excluded). Q: quarter.

Figure 3 shows the number of unique users accessing open notes over each quarter-year, where a sharp increase was observed when open notes went live followed by a gradual decline of 47.8% (–941/1968) from the maximum. Figure 4 excludes COVID-19 assessments from the dataset. Here, a more gradual increase is observed, followed by a slight decline of 7.4% (–74/1000) from the maximum by the end of the study period.

**Table 1:** Notes accessed under each clinical interaction type.

Clinical interaction type	Total documents, n	Unique documents, n
COVID-19	7476	4725
FM^a^, IM^b^, or primary care	3043	2213
Head and neck	1029	593
Hematology and oncology	912	654
Bariatrics	861	674
Gastroenterology	810	430
Cardiology	664	437
Infectious disease	653	474
Unknown	565	343
Rheumatology	475	300
Dental oncology	364	266
Pulmonology	339	209
Telemedicine	281	200
Nutrition	186	165
Occupational medicine	138	90
Orthopedics	119	70
Physiatry	94	63
Urology	73	47
Marijuana clinic	71	53
Oncology	49	30
Neurology	41	26
Pain	40	28
Cardiothoracic surgery	34	12
General surgery	34	23
Accident	31	23
Neurosurgery	1	1
Social worker	1	1
Total	18,384	12,150

^a^FM: family medicine.

^b^IM: internal medicine.

## Discussion

### Principal Findings

The primary purpose of the study was to quantify outpatient open note access and characterize its users at our institution. With 35,273 accounts in the portal, our study highlighted that 13.1% (4615/35,273) of portal registrants viewed their outpatient notes. This is in line with previous studies that found user rates of 13.7% [7] and 16.2% [8]. Some studies have also noted a female bias with respect to patient portal usage [9-12] and note access specifically [7,8], and our data showed that open note access is no different, with 63.4% (2926/4615) of patients being female. However, this number may be more reflective of the entire patient population connected to the portal (female: 20,047/35,273, 56.8%) rather than any sex-specific attributes of open note access. Interestingly, the bias was not as apparent in those aged 0-19 and ≥80 years, who may be managed by a proxy [11], but this was not delineated further in our study. It would be useful to explore whether there were sex disparities in the total number of clinic visits among users who accessed notes to gain further insight into this trend.

This study also uncovered a bimodal distribution in the age of patients that viewed open notes (30s and 50s). This finding is not unique in the literature, and it has been suggested that younger individuals may be more comfortable using the functions of the portal while older individuals are likely to be more medically complex [10] and have more notes to access. Furthermore, the percentage of notes accessed via a mobile device tended to decrease as a function of increasing age, indicating that web-based access of portal function is still a vital part of the way older patients interact with their data. However, this trend was not observed in those aged ≥90 years, who may have younger proxies managing their portal access [11]. Since this subset only consisted of 6 individuals in our study, it is imperative that further research be conducted with individuals within this age group to further give credence to this hypothesis of the possibility of proxy access.

When open notes went live, a sharp increase in the number of users accessing notes was observed, implying a high level of initial interest. The increase in the beginning may also be the result of more patients creating portal accounts and pursuing registration data would have created a useful point of comparison. However, as time evolved, the number of users accessing notes steadily declined by 47.8% (–941/1968). A likely explanation could be the content of the notes themselves: approximately 38.9% (4725/12,150) represented COVID-19 assessments and the falling levels may be due to fewer visits as the pandemic subsided. To further outline this trend, Figure 4 was plotted without COVID-19 assessments. Here, the number of users accessing open notes more gradually increased, reaching a peak in the third quarter of 2022, and ending with a slight decline of 7.4% (–74/1000) from the maximum. This suggests that outside of the pandemic context, user saturation may have been achieved among those interested in viewing open notes. The slight decrease at the end may be indicative of fading interest in open notes, although more data would be needed to establish whether this trend continued after the study period. We hypothesize that it will likely level off to a point below the maximum but still be appreciable, similar to what was observed for other digital tools such as telemedicine [13,14]. Future studies should compare open note users to all active users of the portal and examine factors that would increase user engagement.

### Limitations

This study has several limitations. First, although 12,150 unique documents were accessed in the portal, we identified 1829 (15.1%) duplicates that we retained due to reasons mentioned in the methods. Since we relied on exact time stamp data to identify duplicates, system-related delays between the upload of identical documents to the portal may underestimate this value. In addition, the “clinical interaction type” data are also predicated on the assumption that all providers used the correct note title to document the encounter. Although we expect any deviations from this assumption to be relatively small, the contents of Table 1 may not be a true reflection of reality. This research led to a quality improvement project to improve the upload of documents so that a single note in the EHR corresponded to a single viewable link in the portal with a title reflective of the context. Second, although our analysis reported a 13.1% (4615/35,273) rate of open note use among all portal registrants, the number may be misleadingly low because the denominator included both outpatient and inpatient registrants. The 2 categories are not mutually exclusive but do not completely overlap either. Separating the outpatient from inpatient accounts would have required significantly complex queries with the use of more audit files, which we did not attempt to pursue in this study. Third, the portal likely contained dormant accounts from patients who registered but did not actively use it, and the definition of an “active user” could have multiple meanings [15], which was not taken into consideration during our study. Using a standard definition of active user would have allowed us to better compare open note use to other portal functions such as medication refills or provider messaging. Fourth, we did not pursue account registration data versus time nor the number of clinic visits by open note users, which was beyond the scope of the objectives. Obtaining this information would have allowed a greater understanding of the trends in open note use, and future studies are planned to explore this further. Finally, there may be errors with demographic data when patients register for the portal, such as inputting the wrong birthdate, which we did not quantify, although we expect this number to be small.

### Conclusions

Despite the limitations, our study demonstrates that open note access was largely dominated by viewing COVID-19 assessments, and the number of users accessing their notes has decreased over time as the pandemic receded. Furthermore, female patients and those aged in their 30s as well as 50s viewed more notes than other groups, and web-based access of open notes remains an important modality for older patients.

## Abbreviations

EHR: electronic health record
HIPAA: Health Insurance Portability and Accountability Act
ID: infectious disease
IRB: institutional review board
